# Identification of allergens in *Artocarpus heterophyllus*, *Moringa oleifera*, *Trianthema portulacastrum* and *Syzygium samarangense*

**DOI:** 10.1186/s12948-023-00187-2

**Published:** 2023-08-11

**Authors:** Janitha Iddagoda, Peshala Gunasekara, Shiroma Handunnetti, Chandima Jeewandara, Chandima Karunatilake, Gathsaurie Neelika Malavige, Rajiva de Silva, Dhanushka Dasanayake

**Affiliations:** 1 Institute of Biochemistry, Molecular Biology and Biotechnology, University of Colombo, Colombo, Sri Lanka; 2 Department of Immunology, Medical Research Institute, Colombo, Sri Lanka; 3 Allergy, Immunology and Cell Biology Unit, Department of Immunology and Molecular Medicine, University of Sri Jayewardenepura, Nugegoda, Sri Lanka

**Keywords:** Food allergy, IgE-mediated, Anaphylaxis, Allergens, Jackfruit, Moringa, Horse purslane, Rose apple

## Abstract

**Background:**

It is clinically important to identify allergens in *Artocarpus heterophyllus* (jackfruit), *Moringa oleifera* (moringa), *Trianthema portulacastrum* (horse purslane) and *Syzygium samarangense* (rose apple). This study included 7 patients who developed anaphylaxis to jackfruit (1), moringa (2), horse purslane (3) and rose apple (1). We sought to determine allergens in the edible ripening stages of jackfruit (tender, mature, and ripened jackfruit) and seeds, edible parts of moringa (seeds, seedpod, flesh inside seedpod, and leaves), horse purslane leaves and ripened rose apple fruit. The persistence of the allergens after cooking was also investigated.

**Methods:**

Allergens were identified by clinical history followed by a skin prick test. Protein profiles of plant/fruit crude protein extracts were determined by SDS-PAGE. Molecular weights of the allergens were determined by immunoblotting with patient sera.

**Results:**

A heat-stable allergen of 114 kDa in *A. heterophyllus* which is shared among different ripening stages and seeds was identified. Additionally, 101 kDa allergen in boiled tender jackfruit, 86 kDa allergen in boiled seeds and 80 kDa allergen in boiled mature jackfruit were identified. Five heat-stable allergens of 14, 23, 35, 43, and 48 kDa in *M. oleifera*, 1 heat-stable allergen of 97 kDa in *T. portulacastrum*, and 4 allergens of 26, 31. 60, and 82 kDa in *S. samarangense* were identified.

**Conclusion:**

Novel IgE-sensitive proteins of *A. heterophyllus, M. oleifera, T. portulacastrum* and *S. samarangense* were identified which would be especially useful in the diagnosis of food allergies. The identified allergens can be used in Component Resolved Diagnostics (CRD).

## Background

Food-induced anaphylaxis is a severe multi-system allergic reaction to a food that can be potentially life-threatening [1]. Food is the cause of anaphylaxis in approximately 37.5% of those experiencing anaphylaxis in Sri Lanka and are mainly IgE mediated [2]. *Artocarpus heterophyllus* and *Moringa oleifera* are rare causes of allergy, whereas allergy to *Trianthema portulacastrum* and *Syzygium samarangense* have never been reported [2–7]. In vitro diagnostic methods (ImmunoCAPs) are currently available only for *A. heterophyllus*.

*A. heterophyllus*, jackfruit, a member of the Moraceae family, with edible fleshy bulbs and starchy seeds, is a popular food in Sri Lanka. Tender jackfruit, mature jackfruit, and ripened (raw) jackfruit are the three edible ripening stages. Tender jackfruit, mature jackfruit arils, and jackfruit seeds are often cooked or boiled before consumption, whereas ripened (raw) jackfruit arils are consumed raw. Tender jackfruit (2.0–2.6%), mature jackfruit arils (0.9%), and ripened (raw) jackfruit arils (1.2–1.9%) have more protein than jackfruit seeds (4.7%) [8, 9].

Jackfruit allergy has been previously described as a Bet v 1-related food allergy, causing oral allergy syndrome (OAS)/pollen-food allergy syndrome (PFAS) [4, 10, 11]. A 17 kDa allergen of the PR-10 protein family, a Bet v 1 homologue, has been isolated from jackfruit [10, 11]. Bet v 1 is the major pollen allergen present in silver birch (*Betula verrucose*) pollen [12]. Jackfruit-induced anaphylaxis associated with birch pollen allergy has also been reported [13, 14]. Another case study, however, reported PFAS to jackfruit with no cross-reactivity with Bet V 1 or Bet V 2 [5]. In addition, 2 patients with a history of latex allergy experienced anaphylaxis after consuming dried jackfruit, possibly due to latex food syndrome [15, 16]. We report a patient of Sri Lankan origin without PFAS who developed allergy and anaphylaxis after eating ripened (raw) jackfruit, as well as a mild allergy to boiled mature jackfruit.

Allergens of different ripening stages of jackfruit have not been characterized in previous studies. We sought to determine the allergenicity and shared allergens in all edible ripening stages of jackfruit; mature jackfruit (raw, boiled), jackfruit seeds (raw, boiled), tender jackfruit (raw, boiled) and in ripened (raw) jackfruit.

*M. oleifera* (moringa) is a multi-purpose herb [17], in the family Moringaceae and order Capparales. Boiled moringa seeds, seedpods, flesh inside seedpods and leaves are consumed worldwide. Anaphylaxis following ingestion of young moringa leaves [18], cooked leaves [19], and cooked seed pods [7] have been reported. Furthermore, occupational asthma caused by moringa seed powder has been described, with allergens identified as proteins of 9, 12, 16, 24, 27, 31, 36, 43, 51, 56, and 71 kDa from raw moringa seed extracts [6]. Moringa allergies have previously been reported in Sri Lanka and the United Kingdom as well [2, 3, 20]. Moringa seedpod, flesh inside seedpod and leaf allergens have not been identified. In our study, we report two patients who developed anaphylactic reactions to cooked moringa seedpods (2) and leaves (1). We further continued our study to determine the allergenicity of moringa leaves (raw, boiled), seedpods (raw, boiled), flesh inside seedpods (raw, boiled), and seeds (raw, boiled).

*T. portulacastrum*, also known as horse purslane, desert horse purslane, sarana, heen sarana, black pigweed, or giant pigweed belongs to the family Aizoaceae and the order Caryophyllales. This plant is widely grown in Southeast Asia, tropical America, and Africa, and its cooked leaves are a popular dish, particularly in Southeast Asia and Africa [21]. There are two forms “red horse purslane” and “white horse purslane”, with “white horse purslane” being the most common [16]. Horse purslane has not been reported to cause allergies previously. We report 3 individuals who developed anaphylaxis after ingestion of cooked horse purslane leaves, and proceeded to identify allergens of this plant.

*S. samarangense* (rose apple, jambu, java apple, wax apple, semarang rose apple, wax jambu), a popular tropical and subtropical fruit, is a member of the family Myrtaceae and order Myrtales. There have been no reports of rose apple allergy. However, cloves (*Syzygium aromaticum*), a member of the same genus cause burning skin reactions when applied [22]. Pollen of *Eucalyptus spp,* which belongs to the same family, also triggers allergies [23]. Even though in vitro diagnostic methods (crude ImmunoCAPs) for cloves and eucalyptus are available, allergens in these plant species have not been identified. We report one case of anaphylaxis to rose apple. The allergens of this fruit were determined as the final objective of our study.

## Methods

### Ethics clearance

Ethics clearance for this study was obtained from the Ethics Review Committee, Medical Research Institute, Colombo, Sri Lanka (ERC no: 11/2019).

### Patients and controls

Patients who developed allergy/anaphylaxis following ingestion of jackfruit, moringa, horse purslane and rose apple were recruited. Clinical data were obtained using an interviewer-administered questionnaire. Additionally, the medical records were reviewed to gather additional information. Sensitization to each plant species was confirmed by skin prick testing. Briefly, prick-to-prick testing was done with the culprit food, along with the positive (histamine) and negative (normal saline) controls [24]. Wheel diameter of > 3 mm more than the negative control was regarded as positive. Clinical details are shown in Table 1.

**Table 1:** Details of patients with allergy to *Artocarpus heterophyllus*, *Moringa oleifera*, *Trianthema portulacastrum* and *Syzygium samarangense*

Patients	Age at onset (years)	Gender (male/female)	Type of food allergy	Skin prick test ( ±)	Episodes	Symptoms	Anaphylaxis or not	Other food alllergies
*A. heterophyllus* (jackfruit)								
A	31	Female	Boiled mature jackfruit (kos)	+	Multiple	Mild abdominal pain	No	No
			Ripened (raw) jackfruit (waraka)	+	Multiple	Angioedema, generalized urticaria, throat swelling, loose stools, vomiting, abdominal pain	1 episode of anaphylaxis and multiple episodes of urticaria and angioedema	No
*M. oleifera* (moringa)								
M1	67	Female	Boiled seedpod	+	1	Urticaria, chest pain, abdominal pain	Anaphylaxis	No
M2	5	Male	Boiled seedpod	+	1	Urticaria, angioedema	Anaphylaxis	No
			Boiled leaves	+	2	Urticaria, angioedema rhonchi, rhinorrhea, sneezing, difficulty in breathing	Anaphylaxis	No
*T. portulacastrum* (horse purslane)								
T1	between 25 and 38 [diagnosis—38]	Male	Boiled leaves	+	Multiple	Urticaria, sweating, rhonchi, loss of consciousness, cardiac arrest, chest pain, difficulty in breathing, abdominal pain, vomiting,	Anaphylaxis	No
T2	52	Male	Boiled leaves	+	3	Urticaria, pruritus, loss of consciousness	Anaphylaxis	No
T3	35	Male	Boiled leaves	+ [4 mm]	2	Urticaria, difficulty in breathing, bradycardia, loss of consciousness, cardiac arrest	Anaphylaxis	No
*S. samarangense* (rose apple)								
S	3	Female	Ripened fruit	+ [3 mm]	2	Angioedema, cough, rhonchi, lifelessness	Anaphylaxis	No

Blood samples (5 ml) were collected from patients (jackfruit-1, moringa-2, horse purslane-3, rose apple-1) and healthy controls (n = 3) after obtaining informed written consent. Serum was separated and stored at −20 °C.

### Sample identification, selection and processing

**Fig. 1:**
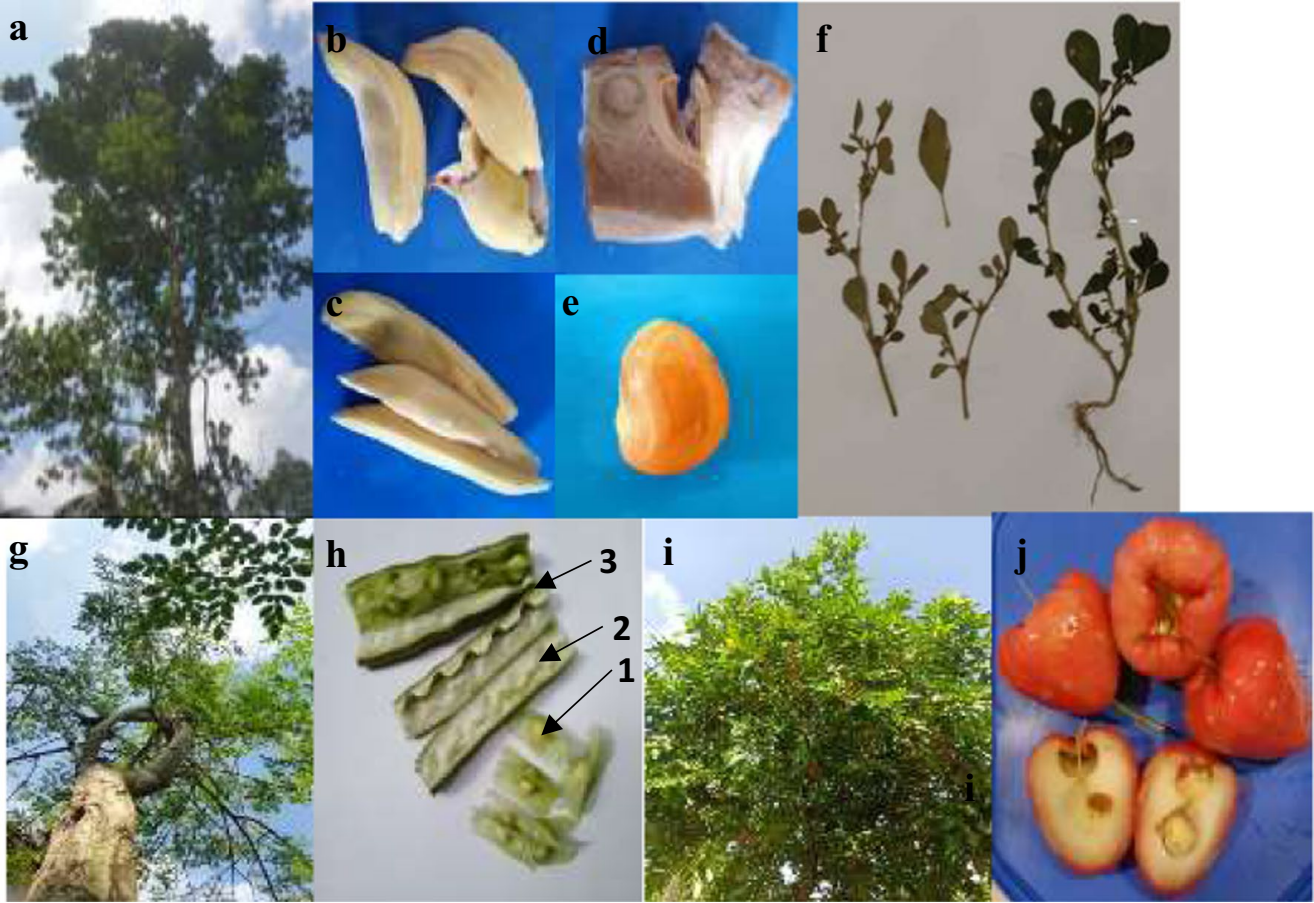
Plant materials used for allergen identification; **a**—*Artocarpus heterophyllus* (jackfruit) **b**—mature jackfruit arils **c**- ripened (raw) jackfruit arils **d**—tender jackfruit **e**—jackfruit seed **f**—*Trianthema portulacastrum* (horse purslane) g—*Moringa oleifera* (moringa) **h**—moringa seedpod (**1**—seeds **2**—pod **3**—flesh) **i**—*Syzygium samarangense* (rose apple) **j**—rose apple fruit

All the plant species were verified from the National Herbarium, Peradeniya, Sri Lanka, according to their morphological characteristics. The edible plant materials of each plant (jackfruit—tender jack fruit, mature jack fruit arils, ripened jack fruit arils and seeds, moringa—seedpod, flesh inside seedpod, seeds, and leaves, ripened rose apple and horse purslane leaves) were collected for further processing (Fig. 1). All the plant materials, except ripened (raw) jackfruit arils and rose apples, are mostly boiled/cooked before consumption. Hence, a part of the collected plant materials was boiled at 100 °C accordingly (tender jack fruit for 1 h, mature jackfruit arils, jackfruit seeds,moringa seedpods, the flesh inside the seedpod, seeds and leaves for 20 min, horse purslane leaves for 10 min) to study their allergenicity after cooking.

### Protein extraction and quantification

Proteins were extracted from each plant material using a plant tissue extraction kit (Abcam, Ab206999) or by preparing plant/fruit crude extracts. The extraction kit was used according to the manufacturer’s instructions and the crude extracts were prepared by crushing each plant material using a mortar and pestle in 0.13 M PBS (pH- 7.2) followed by centrifugation at 7000* g* for 5 min.

Of the two extraction methods tested, the study was continued with the more convenient plant/fruit crude protein extraction method using 0.13 M PBS, since the protein profiles given by both extraction methods were similar. Crude extracts of tender jackfruit (raw, boiled), mature jackfruit arils (raw, boiled), jackfruit seeds (raw, boiled), ripened (raw) jackfruit arils, moringa seedpods (raw, boiled), flesh inside moringa seedpods (raw, boiled), moringa seeds (raw, boiled), moringa leaves (raw, boiled), horse purslane leaves (raw, boiled), ripened rose apple were prepared accordingly for allergen identification. Proteins of each sample were quantified by the Bradford assay using bovine serum albumin (BSA) as a standard.

### Sodium dodecyl sulfate–polyacrylamide gel electrophoresis (SDS PAGE)

Protein profiles of each plant extract were determined by SDS-PAGE in 12% polyacrylamide gels under reducing conditions using a mini protein R- apparatus (Bio-Rad). Before electrophoresis, samples were prepared by mixing with 4X Laemmli sample buffer followed by denaturing at 950 °C for 5 min. The proteins and pre-stained protein markers were separated at 70 V at 4 °C for 2 h for visualization with Coomassie brilliant blue R-250. The molecular weights of the proteins were calculated using a standard curve of pre-stained protein markers.

### Immunoblotting

Proteins separated by SDS-PAGE were transferred to a nitrocellulose membrane at 60 V for 150 min in a mini protein tetra system (Bio-Rad). The membrane was blocked using blocking buffer (5% nonfat milk in PBST [0.05% Tween 20 in PBS]) for 1 h at 4 °C followed by washing for 5 min each with PBST three times. To add primary antibodies, patient sera were diluted at 1:20 in blocking buffer and the membranes were then incubated overnight at 4 °C. After washing 3 times in PBST to remove unbound primary antibodies, the secondary antibody, HRP conjugated goat anti-human IgE antibody (Sigma Aldrich) was diluted in a ratio of 1:1000 in blocking buffer, added to the membranes and incubated for 1 h at 4 °C. Finally, the membrane was washed three times with PBST, each washing step for 5 min and the allergen bands in the membrane were visualized using 4-chloro-1-naphthol (4-CN) as a substrate. The molecular weights of the allergens were estimated using a standard curve of pre-stained protein markers.

## Results

### Clinical features of the patients

All patients (7) in this study experienced anaphylaxis (jackfruit-1, moringa-2, horse purslane-3, rose apple-1). They had clinical features including cutaneous (urticaria, angioedema, pruritus), respiratory (rhonchi, rhinorrhea, cough, sneezing, shortness of breath), cardiovascular (bradycardia, cardiac arrest, chest pain, loss of consciousness), and gastrointestinal (loose stools, abdominal pain, vomiting) symptoms (Table 1).

The patient with jackfruit allergy was a female adult (A) who had multiple episodes of mild abdominal pain after eating boiled/cooked mature jackfruit arils and one episode of anaphylaxis and multiple episodes of urticaria and abdominal pain to ripened (raw) jackfruit arils (Table 1). This patient could consume cooked tender jackfruit. She did not give a history of PFAS or latex allergy.

Both patients (female adult-1 [M1], male child-1 [M2]) with moringa allergy had anaphylaxis to boiled seed pods and one (M2) had 2 episodes of anaphylaxis to cooked leaves (Table 1). Patient M1 could consume moringa leaves without any allergic reactions.

All three patients (T1, T2, T3) with horse purslane allergy were adult males who developed anaphylaxis to cooked leaves. Patient T1 had multiple episodes of anaphylaxis whereas patient T2 and T3 had 3 and 2 episodes of anaphylaxis, respectively (Table 1).

A 3-year-old female (S) who was allergic to rose apple experienced two episodes of anaphylaxis after consuming ripened fruit (Table 1). None of the patients had a history of other food allergies.

### Protein concentrations of the plant protein extracts

Raw plant/fruit extracts showed a higher concentration of proteins than boiled extracts. The estimated protein concentrations are shown in Table 2.

**Table 2:** Estimated protein concentrations and the molecular weights of the proteins identified in SDS-PAGE of *Artocarpus heterophyllus, Moringa oleifera, Trianthema portulacastrum* and *Syzygium samarangene*

Plant/fruit extract	Protein (crude) concentrations used in SDS-PAGE (mg/ml)	Molecular weights (kDa) of the proteins identified in SDS-PAGE
*A. heterophyllus* (jackfruit)		
Seeds [raw]	38	8, 14, 20, 23, 33, 35, 41, 47, 55, 68, 79, 91, 114, 123
Seeds [boiled]	23	8, 14, 20, 23, 35, 41, 47, 55, 68, 79, 91, 114, 123
Mature jackfruit [raw]	36	11, 17, 33, 35, 41, 44, 47, 55, 63, 79, 91, 114, 123, 132, 165
Mature jackfruit [boiled]	24	11, 55, 63, 79, 91
Tender jackfruit [raw]	28	8, 14, 17, 20, 23, 33, 35, 41, 47, 55, 68, 79, 91, 114, 123, 165
Tender jackfruit [boiled]	24	–
Ripened (raw) jackfruit	22	11, 17, 33, 35, 41, 44, 47, 55, 63, 79, 91, 114, 123, 132, 165
*M. oleifera* (moringa)		
Seeds [raw]	40	8, 11, 15, 20, 23, 41, 44, 48, 68, 91, 98, 161
Seeds [boiled]	30	8, 11, 23, 41, 44, 48, 63, 68, 79, 91, 98, 134
Seedpod [raw]	28	11, 14, 16, 25, 31, 33, 39, 41, 43, 44, 64, 68, 73, 79
Seedpod [boiled]	23	–
Flesh [raw]	33	18, 23, 29, 33, 41, 44, 48, 51, 55, 59, 68, 91, 98
Flesh [boiled]	21	–
Leaves [raw]	38	14, 16, 29, 36, 48, 59, 64, 68, 73, 79, 85, 91, 105, 130, 150, 161
Leaves [boiled]	43	–
*T. portulacastrum* (horse purslane)		
Leaves [raw]	19	5, 9, 11, 15, 16, 22, 29, 36, 40, 49, 54, 65, 72, 80, 88, 97, 108
Leaves [boiled]	13	5, 9, 11, 15, 16, 22, 29, 36, 40, 49, 54, 65, 72, 80, 88, 97, 108
*S. samarangense* (rose apple)		
Ripened fruit	18	19, 26, 31, 36, 42, 45, 51, 54, 60, 67, 82, 93, 95

### Proteins identified in SDS-PAGE of plant/fruit extracts

**Fig. 2:**
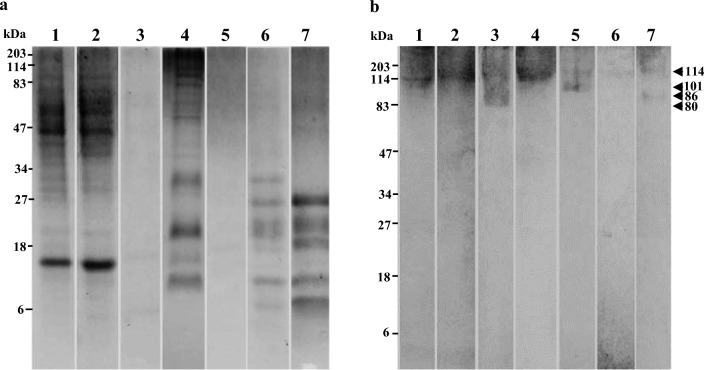
Coomassie-stained SDS-PAGE and Immunoblotting of *Artocarpus heterophyllus* (jackfruit); **a**—SDS PAGE **b**—Immunoblot Lane 1: ripened (raw) jackfruit, Lane 2: raw mature jackfruit, Lane 3: boiled mature jackfruit, Lane 4: raw tender fruit, Lane 5: boiled tender fruit, Lane 6: raw jackfruit seeds, Lane 7: boiled jackfruit seeds

**Fig. 3:**
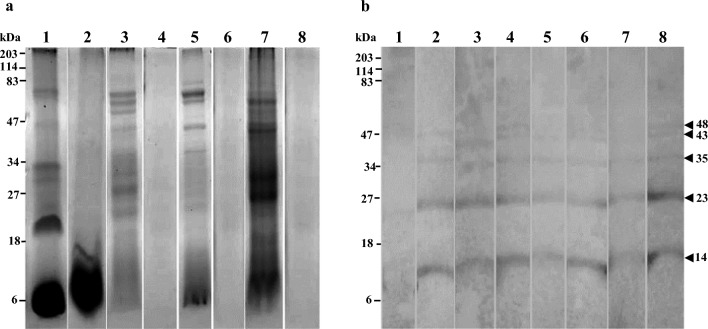
Coomassie-stained SDS-PAGE and immunoblotting^*^ of *Moringa oleifera* (moringa); **a**—SDS-PAGE **b-** Immunoblot Lane 1—raw seeds, Lane 2—boiled seeds, Lane 3—raw seedpod, Lane 4—boiled seedpod, Lane 5—raw flesh inside seedpod, Lane 6—boiled flesh inside seedpod, Lane 7—raw leaves, Lane 8—boiled leaves.^*^ For patient M2

**Fig. 4:**
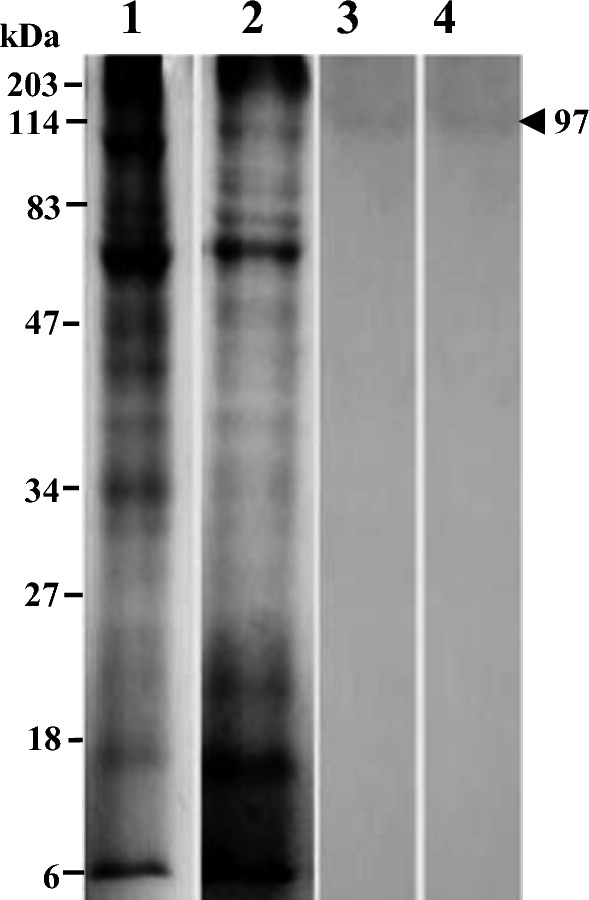
Coomassie-stained SDS-PAGE and Immunoblotting^*^ of *Trianthema portulacastrum* (horse purslane): Lane 1—SDS-PAGE of raw leaves, Lane 2—SDS-PAGE of boiled leaves, Lane 3—Immunoblot of raw leaves, Lane 4—Immunoblot of boiled leaves.^*^ For patient T3

**Fig. 5:**
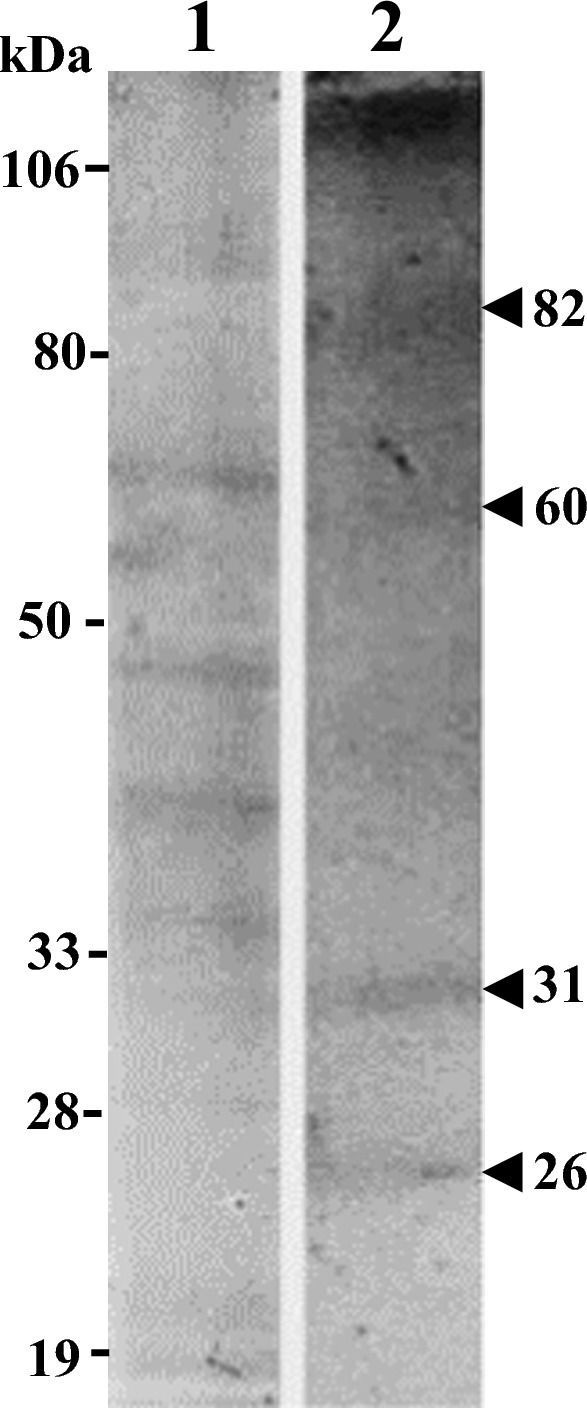
Coomassie-stained SDS-PAGE and Immunoblotting of *Syzygium samarangense* (rose apple): Lane 1—SDS-PAGE**,** Lane 2**—**Immunoblot

Molecular weights of the proteins identified in *A. heterophyllus*, *M. oleifera*, *T. portulacastrum* and *S. samarangense* are shown in Table 2 and Figs. 2a, 3a, 4 and 5.

### Immunoblot analysis of *A. heterophyllus* (jackfruit)

As shown in Fig. 2, one prominent immune reactive band of 114 kDa was identified in raw mature jackfruit ripened (raw) jackfruit. The same band was identified in boiled jackfruit in between a smear ranging from 80 to 200 kDa. The same immune reactive band of 114 kDa was identified in both raw and boiled tender jackfruit and jackfruit seeds as well. Furthermore, bands of 80 kDa, 101 kDa and 86 kDa were also detected in immunoblots of boiled mature jackfruit, boiled tender jackfruit and boiled jackfruit seeds, respectively.

### Immunoblot analysis of *M. oleifera* (moringa)

Five allergens of 14, 23, 35, 43, and 48 kDa were identified in both raw and boiled moringa seedpod, flesh inside seed pod, leaves and boiled seeds. Allergens could not be detected in raw moringa seeds. Only one patient (M2) who was allergic to both seedpod and leaves reacted to these allergens (Fig. 3).

### Immunoblot analysis of *T. portulacastrum* (horse purslane)

One immunoreactive band of 97 kDa was identified in raw and boiled horse purslane leaves. However, only one patient (T3) reacted to this allergen in immunoblots (Fig. 4).

### Immunoblot analysis of *S. samarangense* (rose apple)

Proteins of 26, 31, 60, and 82 kDa were identified as allergens in ripened rose apple (Fig. 5).

With healthy controls, no IgE reactive bands were observed in all plant/fruit extracts.

## Discussion

Allergy to *A. heterophyllus* and *M. oleifera* are rarely reported, whereas *T. portulacastrum* and *S. samarangense* have not been identified as allergens previously. All patients had one or more episodes of anaphylaxis to *A. heterophyllus* (1), *M. oleifera* (2), *T. portulacastrum* (3) or *S. samarangense* (1).

Anaphylaxis to jackfruit has previously been reported in five people (13–16). Three of the reported individuals developed PFAS, one of whom had a history of contact dermatitis to latex [13, 14]. The other 2 individuals were also allergic to latex [15, 16]. The cross-reactivity of Bet v 1 and its homologues (PR 10 proteins) is responsible for the association of birch pollen allergy and PFAS following ingestion of raw fruits from plant families including Rosaceae and Moraceae families [10, 11]. These heat-labile allergens can occasionally cause anaphylaxis [13]. While several fruits have been identified with cross-reactivity to natural rubber latex, the exact allergen responsible for possible cross-reactivity between latex and jackfruit has not been identified.

Our patient did not have a history of pollen allergy and developed anaphylaxis to both raw and heated jackfruit. Therefore, it is unlikely that PR 10 proteins were responsible for the reaction. Furthermore, the patient also did not have latex allergy. Hence, the exact nature of the allergen responsible is uncertain.

A reduced number of proteins were observed with boiled mature jackfruit but no proteins were seen in boiled tender jackfruit using SDS-PAGE. There was no discernible difference in protein profiles between the three ripening phases (raw) of jackfruit. The majority of the proteins (33, 35, 41, 47, 55, 79, 91, 114, and 123 kDa) were found in all stages and seeds. Proteins of 8, 14, 20, 23, and 68 kDa found in seeds were also found in tender jackfruit.

The immunoblot results suggest that 114 kDa protein, the only allergen seen in uncooked stages of the jackfruit may be the causative allergen in jackfruit allergy. Tender jackfruit is normally cooked for a longer time (over an hour) than mature jackfruit and jackfruit seeds (approximately 20 min). The patient's anaphylactic reaction to ripened (raw) jackfruit, minor abdominal pain from boiled mature jackfruit, and lack of symptoms from boiled tender jackfruit may be attributed to heat denaturation and epitope rearrangement of proteins during cooking/boiling. The 80, 101 and 86 kDa allergens observed in boiled mature jackfruit, tender jackfruit and jackfruit seeds respectively, could be degradation products of 114 kDa allergen that occurred during boiling. Nevertheless, these could be different IgE reactive proteins of which new epitopes have been exposed after boiling.

The 17 kDa (Art h 17 kDa) allergen, a Bet v 1 homologue of jackfruit, was not found in the immunoblots of this study [4, 10, 11, 14]. However, SDS-PAGE results showed a protein of 17 kDa in raw mature jackfruit, tender jackfruit and ripened jackfruit. Art h 17 kD is a PR-10 protein, which is heat labile. Conversely, the 114 kDa allergen identified in our study is heat stable. Therefore, the 114 kDa allergen most likely does not belong to the Bet v 1 family. As a result, allergy/anaphylaxis caused by jackfruit consumption may not be caused solely by Bet v 1 allergens. More research with a larger sample size is needed to confirm the presence of the 114 kDa allergen in jackfruit. This study, however, indicates that allergens are present in all edible ripening stages of jackfruit and its seeds, and patients with a history of acute hypersensitivity to jackfruit should avoid both raw or cooked mature jackfruit, tender jackfruit, ripened (raw) jackfruit, and seeds.

Anaphylaxis following ingestion of moringa has been previously described in three patients [7, 18, 19]. Both seeds and leaves of moringa can be responsible for anaphylactic reactions [18]. The allergens responsible for the reactions have not been characterized previously. In our study, only moringa seeds were found to have proteins after boiling, using SDS-PAGE. In contrast, immunoreactive bands (14, 23, 35, 43, and 48 kDa) were found in all four boiled moringa extracts (seeds, seedpod, flesh inside seedpod and leaves). The 43 kDa allergen identified in all moringa extracts tested (except in raw moringa seeds) may be similar to the 43 kDa allergen identified by Poussel et al., 2015, in moringa seed powder in a patient with occupational asthma [6]. The absence of the IgE reactive bands in raw moringa seeds is perplexing. Surprisingly, one of our patients who was allergic to the flesh inside moringa seedpods was able to consume moringa leaves without experiencing any reactions. However, allergens could not be identified in this patient. Conversely, Ichrak, 2022 described a patient who developed anaphylaxis to moringa leaves but without any reactions to seedpod/ flesh inside the seedpod [18]. This suggests that in addition to the identified shared allergens in boiled seeds, raw/boiled seedpod, raw/boiled flesh inside seedpod, and raw/boiled leaves, there may be additional unidentified allergens in moringa edibles. This should be clarified through future investigations. However, our research shows that all raw or cooked edibles of moringa tested could be allergenic (possibly raw seeds as well though it could not be confirmed by immunoblotting) and patients with a history of immediate hypersensitivity to moringa should avoid both raw and boiled moringa seeds, seedpod, flesh inside seed pod and leaves.

To the best of our knowledge, this is the first study to report allergy to horse purslane. Even though all 3 patients had more than 1 episode of anaphylaxis to cooked horse purslane leaves, only one patient reacted to the 97 kDa. The protein concentration of boiled horse purslane leaves used in immunoblots in this study was 13 mg/ml, which was prepared by crushing only 2–3 leaves. The protein concentration used may not have been high enough to get immune reactive bands in immunoblots. Normally, an average person consumes at least 20 g or more of cooked horse purslane leaves per meal, which could contain allergens in higher amounts that could easily induce an allergic reaction. However, the 97 kDa allergen was present in both raw and boiled samples, which suggests that this may be a heat-stable protein. The allergenicity of horse purslane should be investigated further with a larger sample size.

This is also the first study to report *S. samarangense* allergy. Eleven proteins of this fruit were identified in this study, including 4 allergens (26, 31, 60, and 82 kDa). There are two more prevalent Syzygium species with fruits similar to *S. samarangense* which are *Syzygium aqueum* and *Syzygium malaccense*. *S. aqueum* is usually known as watery rose apple, whereas *S. malaccense* is known as Malaysian rose apple. Because three of these species are almost identical, they are all referred to as rose apple or jambu [25]. Hence, allergens of *S. samarangense* are likely to be found in *S. aqueum* and *S. malaccense* as well. However, this should be confirmed through additional research.

All patients in this study have been advised to avoid the consumption of their particular allergenic plants or fruits. Aside from the immunoblotting technique used in this study, there are several other sensitive protein-based approaches for allergen detection and quantification, including mass spectrophotometry, enzyme-linked immunosorbent assay (ELISA) derivatives such as ImmunoCAPs, ISAC, and multiplex assays and biosensors, as well as DNA-based approaches such as PCR and real-time PCR [26].

## Conclusions

A novel heat stable allergen present in all edible ripening stages and seeds of *A. heterophyllus*, 4 heat stable allergens of all edible parts of *M. oleifera*, 1 heat stable allergen of *T. portulacastrum* and 4 allergens in *S. samarangense* were identified. The findings of this study would be particularly useful in the diagnosis of food allergies. Furthermore, consumers should be informed about the allergenicity of these plant species. The amino acid sequences of the detected allergens should be determined in all plant species for further characterization. These allergens should be further explored with a larger sample size. Furthermore, the possibility of using these novel allergens in component resolved diagnosis (CRD) should be considered.

## Data Availability

The datasets used and/or analysed during the current study are available from the corresponding author on upon reasonable request.

## Abbreviations

4-CN: 4-Chloro naphthol
Art h 17: *Artocarpus heterophyllus*-17
Bet V 1: *Betula verrucosa*-1
BSA: Bovine serum albumin
CRD: Component resolved diagnostics
ELISA: Enzyme-linked immunosorbent assay
HRP: Hoarse reddish peroxidase
ISAC: Immuno-Solid phase allery chip
IgE: Immunoglobulin E
kDa: Kilodalton
OAS: Oral allergy syndrome
PBS: Phosphate-Buffered saline
PBST: Phosphate-Buffered saline, tween 20
PCR: Polymerase chain reaction
PR-10: Pathogenesis-related proteins-10
Rf: Retention factor
SDS PAGE: Sodium dodecyl Sulphate-Polyacrylamide gel electrophoresis
